# Repeat corneal graft failure due to graft-to-host herpetic infection

**DOI:** 10.1186/1869-5760-3-24

**Published:** 2013-01-28

**Authors:** Zisis Gatzioufas, Andrea Hasenfus, Balasz Gyongyossy, Evangelos Stavridis, Marlies Sauter, Sigrun Smola, Berthold Seitz

**Affiliations:** 1 Department of Ophthalmology, University of Saarland, Kirrberger Str, Homburg/Saar, Saarland, 66424, Germany; 2 Department of Pathology, University of Saarland, Homburg/Saar, 66424, Germany; 3 Institute of Virology, University of Saarland, Homburg/Saar, 66424, Germany

**Keywords:** Penetrating keratoplasty, Herpes simplex virus, HSV, Corneal graft failure, DNA-PCR, Immunohistochemistry

## Abstract

**Background:**

Herein, we present the case of a young female patient with keratoconus, who was subjected twice to repeat keratoplasty, and each time, she experienced a corneal graft failure.

**Findings:**

Under the suspicion of herpetic eye disease, we administered topical and systemic anti-herpetic treatment after the second repeat keratoplasty. The postoperative course was uneventful, and the corneal graft is clear, until recently. Immunohistochemistry and DNA-polymerase chain reaction were negative for herpes simplex virus-1 (HSV-1) in the host cornea, but they detected HSV-1 in both transplanted corneal grafts, thereby supporting our clinical hypothesis that graft-to-host HSV-1 infection elicited this chain reaction of complications in our patient.

**Conclusion:**

This clinical report illustrates in a unique way the dramatic impact an unsuspected herpetic infection in the corneal graft in cases of keratoplasty may have and underscores the necessity of suspecting and adequately treating these distinct cases.

## Findings

### Introduction

Several authors have reported the occurrence of herpetic keratitis after penetrating keratoplasty in patients with no history of herpetic disease [1-3]. It has been hypothesized that graft-to-host transmission of herpes simplex virus 1 (HSV-1) may cause herpetic keratitis, which is described as ‘newly acquired’ keratitis [2,4]. However, reactivation of a latent HSV-1 infection may also account for persisting corneal epithelial defects or even corneal graft failure after penetrating keratoplasty [5]. Hereby, we present a unique case of repeat corneal graft failure after penetrating keratoplasty, which is associated with graft-to-host HSV-1 infection.

### Case report

**Figure 1:**
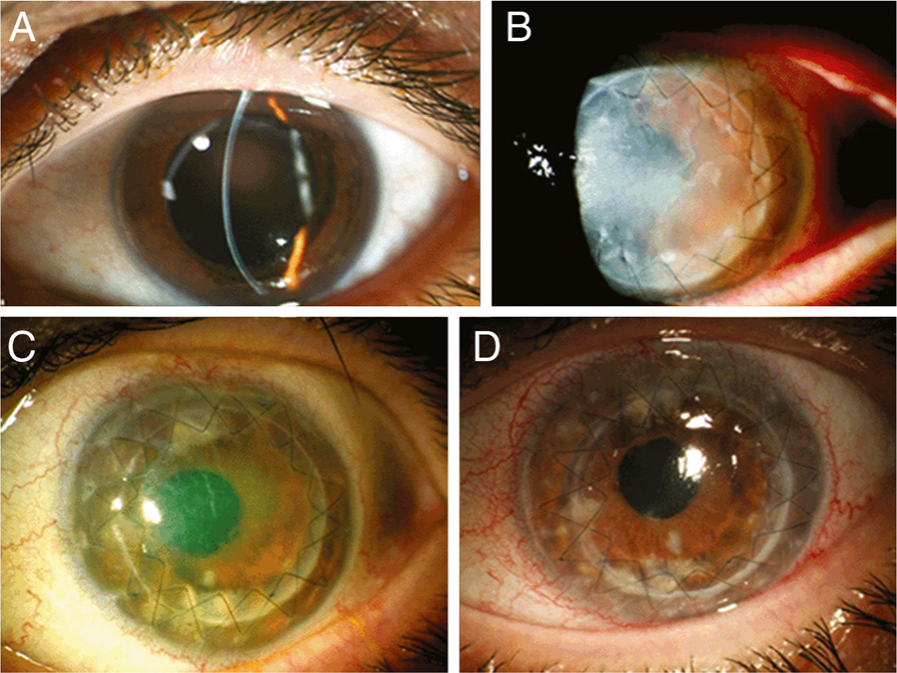
**Slit-lamp examination of the left eye.** (**A**) Corneal irregularity with marked thinning of the central cornea is observed in June 2008. (**B**) Slit-lamp examination of the left eye after penetrating keratoplasty revealed a diffuse cloudiness of the corneal graft with stromal oedema (June 2009). (**C**) Slit-lamp examination of the left eye in November 2009 showed an epithelial defect on the central cornea, which did not heal despite intensive topical therapy with autologous serum. (**D**) The corneal graft is clear after the second repeat keratoplasty, and there are no signs of infection (February 2011).

A 45-year-old female patient presented in our outpatient clinic in June 2008, complaining of gradual deterioration of visual acuity in the left eye (OS) for 1 year. She had no medical history and received no medication. Best-corrected visual acuity (BCVA) was 9/10 in the right eye (OD) and 1/20 OS. Intraocular pressure (IOP) was 14 mmHg OD and 15 mmHg OS. Objective refraction was +2.5/−1.25/55° OD and +1.5/−2.25/45° OS. Slit-lamp examination revealed central corneal irregularity with marked thinning of the paracentral cornea OS (Figure 1A). Scheimpflug examination confirmed this finding (central corneal thickness OS, 482 μm), and a clinical diagnosis of keratoconus was made. After a thorough explanatory conversation, we recommended a penetrating excimer laser-assisted keratoplasty (PKP) OS, and the patient was enrolled on our waiting list.

In December 2008, the patient underwent an uneventful PKP OS. Because of a persisting epithelial defect postoperatively and despite topical treatment with autologous serum, the patient was subjected to amniotic membrane transplantation 2 weeks later. She was discharged with a bandage contact lens and prednisolone eye drops three times a day, ofloxacin eye drops five times a day and artificial tear drops five times a day. BCVA was 10/10 OD and 1/25 OS.

In June 2009, the patient referred to our clinic for regular follow-up examination. Slit-lamp examination revealed a diffuse cloudiness of the corneal graft due to the integrated amniotic membrane, with stromal oedema and multiple Descemet membrane folds (Figure 1B) [6,7]. Since the graft had never been clear after PKP, the diagnosis of primary graft failure was made, and an intensive treatment with corticosteroids was administered (prednisolone eye drops hourly, prednisolone 250 mg intravenously for 3 days on a tapering dose). Despite topical and systemic cortisone therapy, we did not observe a clinical improvement, and the patient was included in our waiting list for a repeat keratoplasty OS. BCVA was 10/10 OD and hand movement OS.

In July 2009, the patient underwent an uneventful repeat PKP OS. On postoperative day 7, the corneal epithelium was healed, and the patient was discharged on a tapering dose of topical corticosteroids (prednisolone eye drops eight times a day, ofloxacin eye drops four times a day for a week, dexpanthenol eye gel five times a day). BCVA was 09/10 OD and 1/20 OS. IOP was 17 mmHg OD and 16 mmHg OS. Follow-up examination was planned in 8 weeks. After 2 weeks, the patient was referred to our emergency clinic with elevation of the IOP OS. IOP was 19 mmHg OD and 31 mmHg OS. Under topical therapy, the IOP was normalized, and upon suspicion of steroid response, the patient was discharged with rimexolone eye drops four times a day, dexpanthenol five times a day and a fixed combination of brimonidine/timolol two times a day. BCVA was 9/10 OD and 4/10 OS.

In November 2009, the patient presented in our clinic with a persisting epithelial defect OS, which did not heal despite intensive topical therapy with autologous serum (Figure 1C). In December 2009, an amniotic membrane transplantation was performed OS. After 4 weeks, the epithelial defect was healed, and BCVA was 1/10 OS.

In October 2010, the patient presented with ocular pain OS. BCVA was 10/10 OD and 1/10 OS. Slit-lamp examination revealed diffuse haze of the corneal graft with prominent stromal oedema due to endothelial decompensation. Anterior chamber examination showed 1+ cells, and no Khodadoust line was observed. IOP was 18 mmHg OD and 36 mmHg OS. The diagnosis of chronic diffuse endothelial graft failure was made, and the patient was commenced on intensive topical and systemic treatment with corticosteroids (prednisolone eye drops hourly, prednisolone 250 mg intravenous for 3 days on a tapering dose). After 1 week, the patient was discharged with prednisolone eye drops eight times a day (on a tapering dose), dexpanthenol five times a day, a fixed combination brimonidine/timolol two times a day and methylprednisolone 80 mg per OS daily (on tapering dose). Nonetheless, clinical findings OS did not improve significantly, and the decision for a second repeat PKP was made.

**Figure 2:**
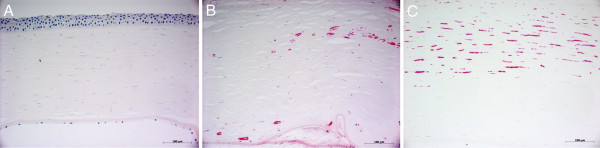
**Immunohistochemical analysis of the host cornea.** (**A**) Immunohistochemical analysis of the host cornea for herpes simplex virus 1 (HSV-1) was negative (×20). (**B**) Immunohistochemical examination of the first corneal graft (after the first repeat keratoplasty) showed a strong signal for HSV-1 (×20). (**C**) Immunohistochemical analysis of the second corneal graft (after the second repeat keratoplasty) also revealed a strong signal for HSV-1 (×20). Scale bar = 100 μm.

In November 2010, the patient underwent a re-repeat PKP for chronic diffuse endothelial graft failure OS. BCVA was 10/10 OD and 1/10 OS. IOP was 15 mmHg OD and 17 mmHg OS. Under the suspicion of herpetic eye disease, we administered, in addition to the usual post-keratoplasty therapy, topical and systemic anti-herpetic treatment (acyclovir 400 mg five times a day for 4 weeks and afterwards two times a day for 12 months, ganciclovir eye gel once a day). Immunohistochemical analysis of the native host cornea was negative for HSV-1 (Figure 2). Polymerase chain reaction (PCR) confirmed the absence of HSV-1 in the host cornea. However, immunohistochemical and PCR analyses of both the first and second corneal graft were positive for HSV-1 (Figure 2). The sensitivity of the applied PCR technique was very high (approximately 98%). The viral load in the first corneal graft was 4 × 10^3^ copies/mL, and the viral load in the second corneal graft was 6 × 10^6^ copies/mL. The postoperative course after the re-repeat PKP was excellent under systemic anti-herpetic treatment, and the corneal graft is clear, until recently (Figure 1D).

### Discussion

Biswas et al. were the first to describe the clinical hypothesis of graft-to-host transmission of HSV-1 [8]. Later on, Remeijer et al. presented clinical data supporting this clinical scenario [9]. Jhanji et al. have recently reported an interesting case series of patients with ‘new-onset’ herpetic eye disease after ocular surgery, suggesting that graft-to-host transmission of HSV-1 may have occurred [4]. However, the clinical impact of graft-to-host herpetic infection is controversial, based on the fact that such a clinical scenario is a rare phenomenon [10].

Our patient had no history of herpetic eye disease. When evaluating the entire clinical course of this case, our patient suffered postoperatively from persisting corneal epithelial defects due to impaired corneal re-epithelialization and experienced one episode of keratouveitis as well as two corneal graft failures. The common etiological factor inducing this chain reaction of ‘unexplained clinical pitfalls’ could have been HSV-1 since the herpetic eye disease is a chameleon regarding the vast variety of clinical expressions and manifestations [5]. Indeed, immunohistochemical analysis and PCR confirmed the presence of HSV-1 in both corneal grafts, but not in the host cornea, thereby supporting our clinical hypothesis that graft-to-host ping-pong HSV-1 infection elicited this chain reaction of complications in our patient. Unfortunately, the corneoscleral ring of the donor tissue was no longer available for HSV DNA-PCR in this patient. Therefore, we cannot definitely exclude a host-derived source of the HSV-1 infection. On the other hand, the second corneal graft may have been salvaged when the patient developed corneal graft haze and IOP elevation if we had performed PCR analysis of an aqueous humour sample for HSV or other viruses, which is an easy, rapid and sensitive method for the confirmation of HSV infection [11]. Instead, the patient was treated more intensively with topical and systemic steroids, which may have potentially aggravated the HSV infection and accelerated the corneal graft failure.

Our clinical suspicion of ‘herpetic eye disease’ was rather delayed, and our patient experienced the dramatic consequences of an untreated herpetic infection in the post-keratoplasty period. This clinical report illustrates in a unique way the dramatic impact an unsuspected herpetic infection on the corneal graft in cases of keratoplasty may have and underscores the necessity of suspecting and adequately treating these distinct cases. The application of aqueous humour analysis either by PCR or by Goldmann-Witmer coefficient analysis for early diagnosis of HSV infection may be of paramount clinical importance in such distinct cases.

All investigations performed in the manuscript were in compliance with the Helsinki Declaration and approved by the Ethics Committee of the University of Saarland/Germany. Written informed consent was obtained from the patient for publication of this report and any accompanying images.

### Conclusion

In conclusion, it is advisable to perform analysis of an aqueous humour sample for detection of potential viral infection in patients with clinically suspected HSV infection after PKP in order to treat early this condition and increase the chances of corneal graft survival.

## Competing interests

The authors declare that they have no competing interests.

## Authors’ contributions

ZG coordinated the study and drafted the manuscript. AH carried out the immunohistochemical examinations. BG participated in the patient examination and follow-up. MS carried out the PCR experiments. SS participated in the PCR experiments and made a critical revision of the manuscript. ES participated in the patient follow-up and provided the slit-lamp photos. BS performed all surgical procedures and made a critical revision of the manuscript. All authors read and approved the final manuscript.
